# Use of Anæsthetic Agents in Ancient China

**Published:** 1853-04

**Authors:** 


### ARTICLE XIX.

### *Use of Anæsthetic Agents in Ancient China.*

Stanislas Julian has found, in examining the Chinese books in the National Library at Paris, the proof that the Chinese have been long acquainted with the use of anæsthetic agents during surgical operations. The extract which he gives is from a book published about the commencement of the sixteenth century, in fifty volumes quarto, and entitled "Kow-Kin-i-tong," "General Account of Ancient and Modern Medicine," and refers to the practice of a celebrated physician, Ho-a-tho, who flourished between the years 220 and 230 of our era. It states, when about to perform certain painful operations, "he gave the patient a preparation of hemp" (hachich,) and that at the end of a few moments "he became as insensible as if he had been drunk or deprived of life." After a certain number of days the patient was cured, without having experienced the slightest pain during the operation. In a subsequent notice he also adds, that the same physician used the hydropathic system as a cure for certain diseases, among others, chronic rheumatism.

*Edinburgh Philosophical Journal.*

